# Impact of breast cancer care pathways and related symptoms on the return-to-work process: results from a population-based French cohort study (CONSTANCES)

**DOI:** 10.1186/s13058-023-01623-6

**Published:** 2023-03-22

**Authors:** Anne-Lise Rolland, Bertrand Porro, Sofiane Kab, Céline Ribet, Yves Roquelaure, Mélanie Bertin

**Affiliations:** 1 Univ. Angers, CHU Angers, Univ. Rennes, Inserm, EHESP, Irset (Institut de recherche en santé, environnement et travail) - UMR_S 1085, SFR ICAT, SIRIC ILIAD, F-49000 Angers, France; 2 Inserm, EHESP, Irset (Institut de Recherche en Santé, Environnement et Travail) - UMR_S 1085, SFR ICAT, SIRIC ILIAD, University Angers, University Rennes, 49000 Angers, France; 3 Département d’Information Médicale, Centre Hospitalo-Universitaire d’Angers, 49100 Angers, France; 4 Department of Human and Social Sciences, Institut de Cancérologie de L’Ouest (ICO), 49055 Angers, France; 5 Unité Cohortes en Population, Inserm, UVSQ, UMS 011, Université Paris Saclay, Université de Paris, Paris, France; 6 Univ Rennes, EHESP, CNRS, Inserm, Arènes - UMR 6051, RSMS - U 1309, F-35000 Rennes, France

**Keywords:** Breast cancer, Care pathways, Return to work, Patterns, Sequence analysis

## Abstract

**Background:**

Breast cancer (BC) treatments and related symptoms may affect return to work (RTW). The objective of this study was to investigate the impact of BC care pathways (timing and sequence of treatments) and related symptoms on RTW.

**Methods:**

The study population included working-age women with BC who were enrolled in the French CONSTANCES cohort from 2012 to 2018. BC treatments, antidepressant/anxiolytic and antalgic drug deliveries (used as proxies of depression and pain, respectively) and statutory sick pay (used to estimate RTW and time to RTW) were assessed monthly using data from the French national healthcare system database. BC care pathways were identified with the sequence analysis method. Cox models with time-dependent covariates were used to investigate the impact of BC care pathways and related symptoms on RTW and time to RTW, after adjusting for age and socioeconomic characteristics.

**Results:**

73.2% (231/303) of women returned to work within 2 years after BC diagnosis. Five BC care pathway patterns were identified: (i) BC surgery only, (ii) BC surgery and radiotherapy, (iii) BC surgery and chemotherapy, (iv) BC surgery and chemotherapy and radiotherapy, and (v) BC surgery and long-term alternative chemotherapy/radiotherapy. The hazards ratios of non-RTW were significantly higher for women who received BC surgery and long-term alternative chemotherapy/radiotherapy and for > 55-year-old women. Time to RTW was significantly longer in women who received chemotherapy (patterns iii to v) and in women with antidepressant/anxiolytic and antalgic drug deliveries.

**Conclusion:**

This study highlights the value of considering the dynamic, cumulative and temporal features of BC care pathways and related symptoms to facilitate the RTW of women with BC.

**Supplementary Information:**

The online version contains supplementary material available at 10.1186/s13058-023-01623-6.

## Background

Breast cancer (BC) is the leading cancer in women worldwide and accounts for 27.8% of all cancer diagnoses in women in Europe [1] and 31% in the USA [2]. Early diagnosis and better treatments have increased the survival rates of patients with BC [3]. As in half of patients, BC is detected before the age of 63 years [4] and retirement age is constantly increasing in high-income countries (e.g., 62 years in France); return-to-work (RTW) and job retention for women with BC are likely to become a major public health issue [5, 6].

RTW after BC is a complex process influenced by many factors [7–12]. BC treatments and related symptoms are considered the strongest prognostic factors of non-RTW [13, 14]. They are usually measured as fixed and independent factors in questionnaires, and the dynamic, cumulative and temporal features of BC care pathways are not taken into account [15–17]. The sequence and combination of BC treatments are in function of the BC stage at diagnosis, the response to treatment (including sides effects) and BC course [12, 14]. Moreover, BC treatments may be associated with short- or/and long-term related symptoms (e.g., fatigue, pain, anxiety, depression) that may change or cumulate over time and influence the RTW process [7, 8, 12]. The determinants and processes implicated in the probability of RTW and the time to RTW also may differ in function of the sequential and cumulative exposure to BC treatments and their duration [5, 9, 18–20]. Consequently, different BC care pathways should be considered when investigating their influence on RTW [17, 19, 21]. However, to our knowledge, only few studies assessed the cumulative impact of different BC treatments on the RTW process, and none considered their sequence and duration [5, 14, 22].

Therefore, the aim of this study was to assess the impact of BC care pathways and their related symptoms on the RTW process after BC diagnosis using a temporal, sequential and cumulative approach and including detailed and objective data on treatments and health status extracted from the French national health insurance system database.

## Methods

### Study population

The study sample was from the CONSTANCES cohort, a population-based prospective cohort study that included ~ 220,000 volunteers aged from 18 to 69 years at 21 health examination centers throughout France between 2012 and 2020 [23]. At inclusion, a health examination was performed, and self-report questionnaires were given to participants to collect sociodemographic, lifestyle, socio-professional data and medical history. The follow-up included self-report questionnaires (filled in at home) once per year and a health examination every 4 years [23]. The CONSTANCES cohort was linked to the national health insurance system database [24] (SNDS) to obtain additional data on BC diagnosis, BC treatments, other treatments reimbursed to the patients due to BC-related problems, such as anxiolytic/antidepressant and antalgic drugs, and statutory sick pay from January 2012 to December 2019. Women with breast cancer were identified in the SNDS from hospitalization records (principal or related diagnoses with two ICD-10 codes = C50—Malignant neoplasm of breast, D05—Carcinoma in situ of breast) and/or long-term chronic disease benefits (ICD-10 = C50, D05). The quality of the BC’s identification in the SNDS was previously tested using medical records for a subsample of 265 cases, resulting in a high positive predictive value (PPV = 92% 95% CI [88–95%]).

**Fig. 1:**
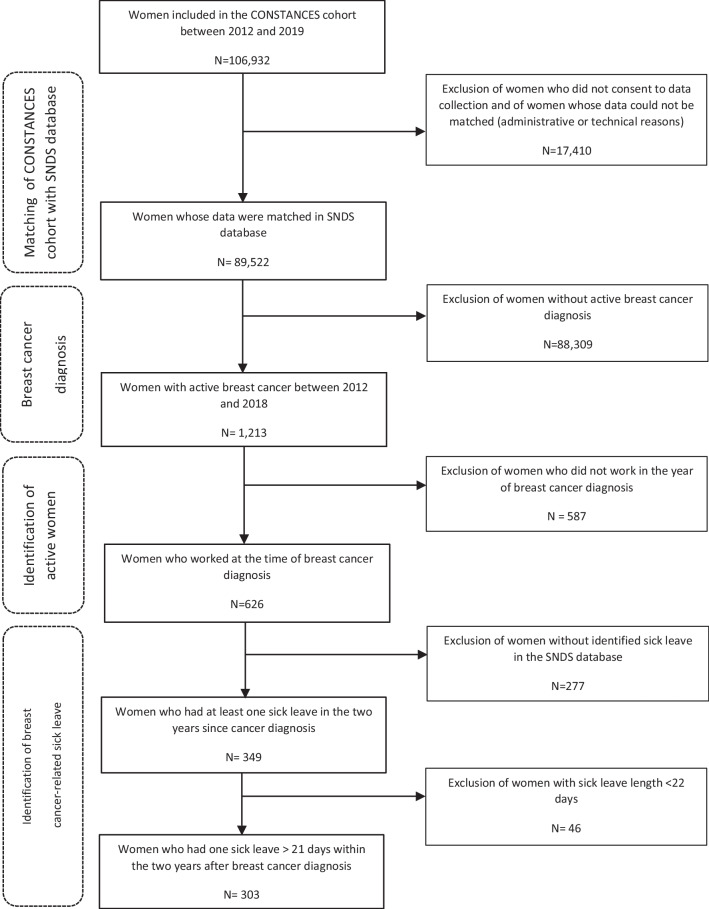
Flowchart of the study population selection SNDS: French national healthcare system database

Analyses were restricted to women from the CONSTANCES cohort who were working at the time of BC diagnosis (from 2012 to 2018) and who gave their informed consent for SNDS data collection (Fig. 1). BC cases were predominantly diagnosed the year of their inclusion in the CONSTANCES cohort or the year before (*n* = 391/626, 62.5%).

### Identification of RTW and time to RTW

RTW and time to RTW were estimated using data on sick leaves (SL) identified from the daily sick pay in the SNDS. A woman with BC was considered as having gone back to work when her daily sick pay was stopped. No distinction between partial- and full-time RTW was made in the statistical analysis. Women on therapeutic partial sick leave were considered to have gone back to work. Patients were considered to have a BC-linked SL if it lasted more than 21 days to avoid SL due to other occasional health issues. The time to RTW was calculated from the first day of the > 21-day-long SL to the last day of the sick pay in the 2 years after the BC diagnosis. Civil servants and self-employed women, for whom sick pay data are not available in the SNDS, were excluded from the statistical analysis on RTW and time to RTW (Fig. 1).

### Identification of BC care trajectory patterns

BC care pathways were identified using the sequence analysis method that allowed to consider the temporal order of successive BC treatments [16, 17, 25, 26]. Each woman was represented by a sequence of states over time that corresponded to different BC treatments she received each month, from the first month of BC treatment identified in the SNDS (*t*_0_) to month 24 after treatment initiation (*t*_24_). Seven states were included: the five BC treatment types identified in the SNDS (mastectomy, breast-conserving surgery, chemotherapy, radiotherapy, and breast reconstructive surgery), the chemotherapy and radiotherapy state (i.e., women who underwent alternatively these two treatments within the same month), and treatment-free months. Axillary lymph node (LN) dissection and hormone therapy also were recorded in the SNDS, but they were not included in the BC treatment sequences. Indeed, axillary LN dissection is usually performed during breast surgery, and hormone therapy is mainly used to prevent BC recurrence and is prescribed over a long period of time (5 and up to 10 years after diagnosis), usually when women are already back at work.

Then, the optimal matching algorithm [27] was used to assess the dissimilarity among the women’s sequence of treatments. Treatment sequences were clustered into homogeneous patterns by hierarchical clustering using the Ward criterion. The appropriate number of clusters was chosen on the basis of the dendrogram and the histogram of the gaps in inertia, and also according to the clinical recommendations for BC care and statistical constraints (i.e., enough patients in each cluster) (Additional file 1: Fig S1).

### Determinants of RTW

Sociodemographic characteristics (age, education level, household income, household composition, occupational category) were collected from the CONSTANCES self-report questionnaires filled in by the patients at inclusion or during the follow-up (in function of the BC diagnosis date). Information on BC treatments (breast surgery, axillary LN dissection, chemotherapy, radiotherapy, reconstructive surgery) and reimbursement of anxiolytic/antidepressant and antalgic drugs (used as a proxy of depressive symptoms and pain in the 2 years after BC diagnosis) were obtained monthly from the SNDS for the 2012–2019 period (Table 1).

**Table 1:** Sociodemographic characteristics at diagnosis, BC treatments and drug deliveries in the group of active women at BC diagnosis (from the CONSTANCES cohort, 2012–2018; *n* = 626)

	*N*	%
Sociodemographic characteristics		
Age (years) median(IQR)	49.1	44.0–54.3
*Household composition*		
In couple without < 18-year-old children	241	38.5
In couple with < 18-year-old children	170	27.2
Single without < 18-year-old children	161	25.7
Single with < 18-year-old children	54	8.6
*Household income*^*a*^		
Low income	133	21.2
Middle income	253	40.4
High income	240	38.3
*Education level*		
Primary and secondary education	248	39.6
Higher education	378	60.4
*Occupational category at BC diagnosis*		
Intellectual professionals/managers	144	23.0
Employees/clerks	252	40.3
Intermediate profession/technicians	207	33.1
Skilled/unskilled manual workers	23	3.7
BC treatments and drug deliveries^b^		
*Total mastectomy*		
No	467	74.6
Yes	159	25.4
*Breast-conservative surgery*		
No	124	19.8
Yes	502	80.2
*Axillary lymph node dissection*		
No	264	42.2
Yes	362	57.8
*Breast reconstruction*		
No	530	84.7
Yes	96	15.3
*Chemotherapy*		
No	342	54.6
Yes	284	45.4
*Radiotherapy*		
No	346	55.3
Yes	280	44.7
*Hormone therapy*		
No	198	31.6
Yes	428	68.4
*Antidepressant/anxiolytic drug delivery*		
No	243	38.8
Yes	383	61.2
*Antalgic drug delivery*		
No	229	36.6
Yes	397	63.4

*BC*, breast cancer; *IQR*, Interquartile range

^a^Low income: < 1,500€ if one contributor or < 2,800€ if two or more contributors in the household; Middle income: between 1,500€ and 2,000€ if one contributor or between 2,800€ and 4,100€ if two or more contributors; High income: ≥ 2,000€ if one or ≥ 4,100€ if two or more contributors in the household

^b^Each BC treatment or drug was delivered at least once within the two years after BC diagnosis (yes/no)

### Statistical analysis

BC care (BC pathways, antidepressant/anxiolytic and antalgic drug deliveries) and sociodemographic characteristics were described for all women who were working at the time of BC diagnosis (*n* = 626, Fig. 1).

The median follow-up time was estimated by the reverse Kaplan–Meier method [28].

Multivariate Cox models with time-dependent covariates were used to assess the influence of BC treatments on the probability to return to work. Time-dependent variables were: BC treatments (except for breast reconstruction due to the limited number of women in the cohort who received this treatment) and anxiolytic and antalgic drug deliveries (measured monthly). Sociodemographic variables, measured only at BC diagnosis, were considered as fixed variables and were selected from a limited number of variables available in the CONSTANCES questionnaire that were previously described as potential predictors of RTW and/or time to RTW [7, 8, 10, 14, 22]. For each outcome, two multivariate models were performed. In the first multivariate model (model A), each BC treatment was treated as an independent variable, whereas in the second model (model B), the BC care pathway patterns identified by sequence analysis were used instead of the individual BC treatments.

The proportional hazards assumption was tested for the fixed variables included in the Cox models using the Schoenfeld residuals analysis and the Hosmer–Lemeshow test.

Statistical significance was defined by a *p* value < 0.05. All statistical analyses were performed with the R statistical software, version 4.0.2, using the packages 'survival,' 'survminer' and TraMineR'[25].

## Results

### Patient selection

Data for 89,522 of the 106,932 women included in the CONSTANCES cohort between 2012 and 2019 could be linked to SNDS database. In the group with matched data, 1,213 women received a diagnosis of BC between 2012 and 2018 among whom 626 were working at diagnosis time. SL data were available in the SNDS for 349/626 active women, and a SL > 21 days within the two years after BC diagnosis was found for 303 of these women (Fig. 1).

### Description of the study population

The median age at diagnosis was 49.1 years (IQR 44.0–54.3). Among the 626 active women at BC diagnosis, 80% underwent breast conservative surgery, 25.4% total mastectomy, 57.8% axillary LN dissection, 45.4% chemotherapy and 44.7% radiotherapy. Moreover, 68.4% of active women received hormone therapy, and 61% and 63.4% had at least one anxiolytic and antalgic drug delivery within the two years after BC diagnosis, respectively (Table 1).

### Description of BC care pathway patterns

**Fig. 2:**
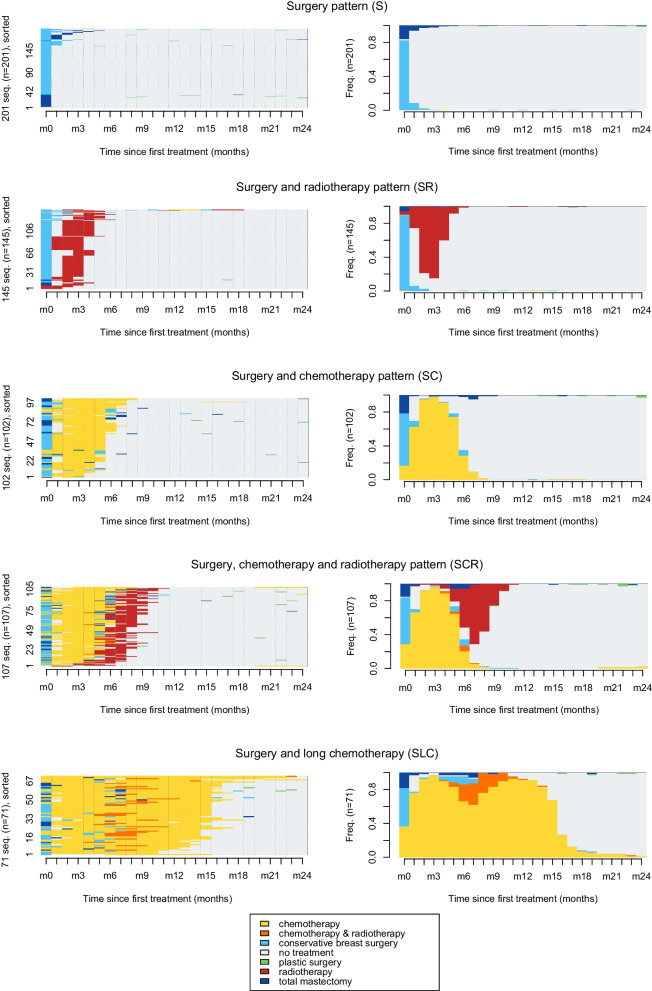
State distribution plots (left panels) and individual sequences of BC treatments (right panels) from treatment initiation to month 24 using the five BC care pathway patterns identified. A color is attributed to each treatment or combination of treatments (e.g., yellow for chemotherapy, dark blue for total mastectomy) as described in the legend

BC care pathways in the two years after the first treatment were identified for the 626 women working at BC diagnosis. Five patterns were determined (Fig. 2): “Surgery” (S, *n* = 201) when treatment was limited to curative BC surgery; “Surgery and Radiotherapy” (SR, *n* = 145) when BC surgery was followed by radiotherapy only; “Surgery and Chemotherapy” (SC, *n* = 102) when BC surgery was followed by chemotherapy only; “Surgery, Chemotherapy, and Radiotherapy” (SCR, *n* = 107) when BC surgery was followed by chemotherapy and radiotherapy (mainly in this order); and “Surgery and Long Chemotherapy” (SLC, *n* = 71) when surgery was followed by a long-term chemotherapy. A part of BCS included in the SLC pattern also underwent an alternating of radiotherapy and chemotherapy sessions and treatment within their care pathways.

*The state distribution plots (right panels) show the percentage of active women with BC who underwent each treatment in the 24 months after treatment initiation. For example, among women in the SLC group, 37% underwent chemotherapy, 45% conservative surgery, 18% total mastectomy at month 1 of treatment, and 76% underwent chemotherapy and 24% a combination of chemotherapy and radiotherapy at month 9 after treatment initiation.*

*The left panels show each BC care trajectory as a sequence of colored lines corresponding to the sequence of treatments received. For example, a strip of light blue and red represents the BC care trajectory of a woman who underwent conservative breast surgery followed directly by radiotherapy. This trajectory is one of the 145 BC care pathways in the SR pattern.*

### RTW and time to RTW

**Fig. 3:**
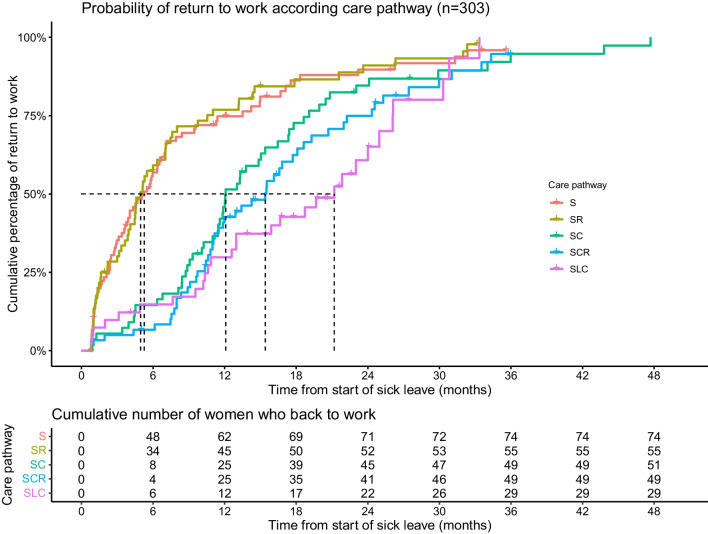
Cumulative incidence of return to work according to the BC care pathways (*n* = 303 active women with BC). S: ‘Surgery’; SR: “Surgery and radiotherapy’; SC: “Surgery and chemotherapy”; SCR: “Surgery, chemotherapy and radiotherapy”; SLC: “Surgery and long chemotherapy”

The median SL duration for the 303 women with SL > 21 days was 9.8 months (mean 11.6, IQR 3.7–15.1, Additional file 1: Table S1) and the median follow-up of the cohort was 22.6 months (Fig. 3). The RTW rate was 33.0% (95%CI 27.7–38.3), 55.8%, (95%CI 50.2–61.4) % and 69.3%, (95%CI 64.1–74.5), 73.2%, (95%CI 71.4–81.0), respectively, at 6 months, 12 months, 18 months and 24 month after BC diagnosis (Fig. 3). The time to RTW varied according to different BC care pathway patterns and increased with the complexity and duration of BC treatments (p < 0.001) (Fig. 3 and Additional file 1: Table S1).

### Association between BC treatments, anxiolytic and antalgic drug deliveries, sociodemographic variables and the probability of RTW

Table 2 presents the associations between probability of RTW and BC treatments, anxiolytic and antalgic drug deliveries, and sociodemographic variables (*n* = 303). The occupational category was the only fixed variable that did not meet the proportional hazards assumption. Therefore, the models were stratified on occupational categories, although in the univariate analysis using the Kaplan–Meier curves and log-rank test, occupational categories were related to the probability of RTW (Additional file 1: Fig S2).

**Table 2:** Associations the between probability of return to work and BC treatments, anxiolytic and antalgic drug deliveries, and sociodemographic variables (*n* = 303)

	N (%)	Model A^a^	p^c^	Model B^b^	p^c^
		Adjusted HR (95%CI)		Adjusted HR (95%CI)	
*Breast surgery*^*d*^					
No	9 (3.0%)	Reference	–		
Yes	294 (97.0%)	0.08 (0.02–0.39)	0.002		
*Chemotherapy*					
No	147 (48.5)	Reference	–		
Yes	156 (51.5)	0.08 (0.03–0.21)	< 0.001		
*Radiotherapy*					
No	166 (54.8)	Reference	–		
Yes	137 (45.2)	0.36 (0.19–0.67)	0.001		
*Axillary lymph node dissection*					
No	106 (35.0)	Reference	–		
Yes	197 (65.0)	0.16 (0.01–1.97)	0.152		
*Hormone therapy*					
No	88 (29.0)	Reference	–		
Yes	215 (71.0)	0.88 (0.62–1.24)	0.455		
*BC care pathways*					
Pattern S	86 (28.4)			Reference	–
Pattern SR	61 (20.1)			1.65 (0.82–3.31)	0.160
Pattern SC	55 (18.2)			0.71 (0.39–1.32)	0.282
Pattern SCR	60 (19.8)			0.76 (0.41–1.39)	0.371
Pattern SLC	41 (13.5)			0.56 (0.31–1.01)	0.056
*Antidepressants/anxiolytics*					
No	104 (34.3)	Reference	–	Reference	–
Yes	199 (65.7)	0.70 (0.51–0.97)	0.032	0.80 (0.58–1.10)	0.171
*Antalgic drugs*					
No	101 (33.3)	Reference	–	Reference	–
Yes	202 (66.7)	0.86 (0.66–1.14)	0.296	0.78 (0.60–1.02)	0.065
*Age (years)*					
20–44	93 (30.7%)	Reference	–	Reference	–
45–49	78 (25.7%)	0.92 (0.55–1.53)	0.744	0.80 (0.47–1.35)	0.400
50–54	74 (24.4%)	1.17 (0.70–1.96)	0.547	1.06 (0.63–1.79)	0.827
≥ 55	58 (19.1%)	0.51 (0.27–0.97)	0.039	0.44 (0.23–0.83)	0.011
*Household income*^*e*^					
Low income	91 (30.0)	Reference	–	Reference	–
Middle income	124 (40.9)	1.21 (0.79–1.86)	0.377	1.10 (0.70–1.74)	0.672
High income	88 (29.0)	2.88 (1.33–6.22)	0.007	2.45 (1.29–5.65)	0.008
*Household composition*					
In couple without < 18-year-old children	122 (40.3)	Reference	–	Reference	–
In couple with < 18-year-old children	75 (24.8)	0.95 (0.55–1.66)	0.868	0.87 (0.50–1.54)	0.637
Single without < 18-year-old children	77 (25.4)	0.67 (0.40–1.14)	0.142	0.65 (0.38–1.12)	0.122
Single with < 18-year-old children	29 (6.6)	0.64 (0.28–1.45)	0.286	0.53 (0.23–1.19)	0.121
*Education level*					
Primary and secondary education	151 (49.8)	Reference	–	Reference	–
Higher education	152 (50.2)	0.99 (0.61–1.60)	0.958	0.92 (0.57–1.46)	0.715

BC, breast cancer; S: “Surgery”; SR: “Surgery and radiotherapy”; SC: “Surgery and chemotherapy”; SCR: “Surgery, chemotherapy and radiotherapy”; SLC: “Surgery and long chemotherapy”

^a^Cox model stratified on occupational categories that included BC treatments and drug deliveries during sick leave as time-dependent variables and sociodemographic variables as fixed variables

^b^Cox model stratified on occupational categories that included BC treatments and drug deliveries during sick leave as time-dependent variables, sociodemographic variables and BC care trajectory patterns as fixed variables

^c^Log-rank test

^d^The two breast surgery types (total mastectomy and breast-conservative surgery) were combined into a single “breast surgery” variable due to limited number of women who underwent total mastectomy

^e^Low income: < 1,500€ if one contributor or < 2,800€ if two or more contributors in the household; Middle income: between 1,500€ and 2,000€ if one contributor or between 2,800€ and 4,100€ if two or more contributors in the household; High income: ≥ 2,000€ if one or ≥ 4,100€ if two or more contributors in the household

In model A, women who underwent breast surgery (HR = 0.08, 95% CI 0.02–0.39), chemotherapy (HR = 0.08, 95% CI 0.03–0.21) and radiotherapy (HR = 0.36, 95% CI 0.19–0.67) were at higher risk of non-RTW. The delivery of antidepressants/anxiolytics was a significant predictor of non-RTW (HR = 0.70, 95%CI 0.51–0.97). In model B, women who received SLC were at higher risk of non-RTW (HR = 0.56, 95% CI 0.31–1.01). In both models, high household income was a RTW facilitator, whereas age > 55 years was a RTW barrier.

## Discussion

This study highlights the importance to assess BC care pathways and related symptoms through a temporal, sequential and cumulative approach and to investigate their independent effects on the probability of RTW. Previous studies already reported the strong impact of BC treatments on RTW [5, 7, 9, 14, 18, 19]. However, very few studies assessed the effects of multimodal treatments and targeted therapies on the probability of RTW [5, 14], and these associations were not significant when the related symptoms were included in the multivariate models. In our study, women who underwent breast surgery, chemotherapy and radiotherapy remained at higher risk of non-RTW even after adjustment for related symptoms (i.e., pain and anxiety and/or depressive symptoms). Conversely, in multivariate models, the risk decreased for axillary LN dissection (Additional file 1: Table S3), possibly due to its association with BC surgery (92% of axillary LN dissections were performed during breast surgery in our study, data not shown) and with antalgic drug deliveries. Axillary LN dissection was previously associated with shoulder function impairment that may lead to chronic pain and complaints [29, 30]. Therefore, adjustment for drug deliveries is likely to be a mediator between axillary LN dissection and RTW, and this could partly explain the non-significant results in multivariate models.

Besides the independent effect of each BC treatment on RTW, we identified five BC care pathway patterns that better reflect BC multimodal management, their order and duration in the two years after diagnosis. The median SL duration was the longest in the BC care pathways with the highest multimodality and longest treatment duration. However, only the SLC pattern (i.e., long-term alternative chemotherapy and radiotherapy) was identified as a barrier to RTW in the two years after BC. Although previous works showed that multimodal therapies, including targeted therapies, affect RTW [5, 14, 22], our study goes further by underlying the importance of considering the temporal and sequential aspects of BC care pathways on the probability of RTW, in addition to their cumulative and multimodal characteristics.

We also investigated the impact of BC/treatment-related symptoms, such as chronic pain and anxious and/or depressive symptoms, using the monthly deliveries of antalgic and antidepressant/anxiolytic drugs to capture their temporal variations in the two years after diagnosis and their influence on RTW [15]. Consistent with the literature [7, 14, 20, 30, 31], our study suggested that the risk of non-RTW was significantly higher in women with more frequent and long-term antidepressant/anxiolytic drug deliveries, independently of their BC care pathway. Similarly, the probability of non-RTW was higher (not statistically significant) in women with more frequent and long-term antalgic drugs deliveries. This is in agreement with previous studies suggesting longer time to partial RTW in women with body or shoulder function impairment due to BC treatment [5, 30].

Although socioeconomic variables and age were considered as confounding factors in our models, our results suggested that they might have a differential impact on the probability of RTW and time to RTW. As previously reported [14], older age (> 55 years) was a barrier to RTW, but did not delay work resumption. This may be explained by the possibility of early retirement arrangements in France that may deter RTW in women close to the legal retirement age. As previously suggested [14], women with high income, professionals and managers were more likely to return to work and more rapidly. Single women with < 18-year-old children were the first to return to work, possibly due to additional socioeconomic and emotional burden of single mothers with BC due to low financial support in their household. Conversely, time to RTW was longest for manual workers (Additional file 1: Fig. S2), possibly due to poorer working conditions that require greater employers’ working adjustments and support to adjust to the work rehabilitation needs of women with BC [8, 11, 32, 33].

### Strengths and limitations

This study presents several strengths explained by the quality of the data collected, particularly their temporal and sequential features, using two complementary statistical methods. First, the nationwide CONSTANCES cohort was linked to the SNDS database to obtain exhaustive and dated data on BC treatments, hospital stays, drug deliveries and daily sick pay in the two years after BC diagnosis, thus avoiding self-reported times, memorization bias and attrition. Two different and complementary statistical methods were used to take into account the temporal and sequential features of BC treatments and their related symptoms over time. They gave similar results underlining their robustness. In the literature, the most frequently used models to study RTW are logistic regressions [9, 10, 14, 20, 22, 34] and classical Cox models that do not take into account this temporality and treatment sequences [5, 31]*.* Instead, a Cox model was chosen in which each BC treatment and drug delivery were treated as time-dependent variables that allowed considering their temporal variations. Furthermore, the sequence analysis method was used to consider the cumulative, order and sequence of different treatments. This method identified five BC care pathway patterns.

Our study presents several limitations. First, we were not able to take into account competitive events, such as retirement, unemployment and disability pension, in our cox models due to the limited number of BCS within each of these situations. Second, reimbursement of anxiolytic/antidepressant and antalgic drugs were used as proxies of depressive symptoms and pain, respectively, in the two years after BC diagnosis. However, these reimbursements could also be related to other physical or mental health issues diagnosed before BC or after BC diagnosis. Third, agricultural and self-employed workers were not included in the CONSTANCES cohort as data on daily sick pay for civil servants are not available in the SNDS. This could have affected the non-RTW rate and the mean duration of sickness absence. However, the percentage of women who did not return to work in the two years after BC diagnosis was similar to the rate reported by a previous French national population-based survey (26.8% versus 25%) [35]. The mean SL duration after BC diagnosis (11.6 months, Additional file 1: Table S1) also was similar to what reported by Fantoni et al. [10] in France and by Balack et al. [5] in the Netherlands (11.5 months). Furthermore, only women with BC and SL > 21 days were included to avoid SL not due to BC. The SL length threshold used in previous studies varied from 14 to 30 days [20, 22]. In our study, women with only short SL (≤ 21 days) received shorter and less complex BC treatments and had less often anxiolytic/antidepressant drug deliveries than women with longer SL (Additional file 1: Table S2). Fourth, the five BC care pathway patterns identified in our study mirror the BC types and treatment protocols, thus minimizing the lack of information on BC stage and targeted therapies [14]. Although hormone therapy and axillary LN dissection are part of BC management, they were not included in the BC care pathways for several reasons. Particularly, most women start hormone therapy when they are already back to work (e.g., 2/3 of hormone therapies were prescribed on year 3 after BC diagnosis in our study) and axillary LN dissections were mainly performed during breast surgery (data not shown).

Finally, BC care pathways were identified only based on BC treatment sequences over time without considering the importance of care and resource organization and coordination [36, 37]. The role of occupational physicians [38], general practitioners [39], breast cancer specialists [40] and of supportive care (i.e., physiotherapists, psychologists, dieticians) after BC diagnosis should also be taken into account to better determine their quantitative and qualitative effect on the RTW process.

## Conclusions

Although most working-age women with BC return to work once they finished their treatments, this study emphasizes the necessity to consider the holistic and sequential aspect of BC care trajectories and related symptoms to better identify women at risk of non-RTW or of longer time to RTW.

## Supplementary Information

**Additional file 1.** Supplementary tables and figures.

## Data Availability

The CONSTANCES cohort and data from the National Health Insurance system are not publicly available as it contains sensitive information. To access the CONSTANCES cohort, a request for extraction must be made to the CONSTANCES team at the UMS 011—Unité cohorts épidémiologiques en population- Université de Versailles Saint-Quentin en Yveline. The CONSTANCES team require a scientific project assed by a scientific committee and a ethical approval to access the data.
