# Supplementary material for: Impact of breast cancer care pathways and related symptoms on the return-to-work process: results from a population-based French cohort study (CONSTANCES)
Source: Breast Cancer Res. 2023 Mar 22;25:30. doi: 10.1186/s13058-023-01623-6 (PMC10031867; doi:10.1186/s13058-023-01623-6)
Supplement: Supplementary file 1 — Additional file 1. Supplementary tables and figures. [file 13058_2023_1623_MOESM1_ESM.docx]

**Supplementary materials**

**Supplementary tables**

**Table S1**. Characteristics at BC diagnosis, treatments in the first 24 months after diagnosis and sick leave in function of the care pathway pattern

**Table S2.** Characteristics and treatments of women with a sick leave identified in the SNDS

**Table S3:** BC treatments associated with the likelihood of return to work, univariate analyses and after adjustment for other BC treatments only (model 1), for BC treatments and drug deliveries (model 2), for BC treatments, socioeconomic characteristics, and age (model 3) (n=303)

**Table S4:** BC care pathway patterns associated with the likelihood of return to work, univariate analyses, and after adjustment for drug deliveries only (model 1), for socioeconomic characteristics and age (model 2) (n=303)

**Table S5:** BC treatments associated with time to return to work, univariate analyses, and after adjustment for other BC treatments only (model 1), for BC treatments and drug deliveries (model 2), for BC treatments, socioeconomic characteristics, and age (model 3) (n=258)

**Table S6:** BC care pathways patterns associated with time to return to work, univariate analyses, and after adjustment for drug deliveries only (model 1), and for socioeconomic characteristics and age (model 2) (n=258)

**Supplementary Figures**

**Fig S1**: Dendrogram showing the hierarchical clustering of different BC treatment sequences using the optimal matching distances, Ward method**.**

**Fig S2.** Cumulative incidence of return to work according to the occupational category.

**Table S1. Characteristics at BC diagnosis, treatments in the first 24 months after diagnosis and sick leave in function of the care pathway pattern**

|  | **All women**  **N=626** | | **Pattern S**  **N=201** | | **Pattern SR**  **N=145** | | **Pattern SC**  **N=102** | | **Pattern SCR**  **N=107** | | **Pattern SLC**  **N=71** | | | | | | | | | **p^a^** | | | | | | |  |
| --- | --- | --- | --- | --- | --- | --- | --- | --- | --- | --- | --- | --- | --- | --- | --- | --- | --- | --- | --- | --- | --- | --- | --- | --- | --- | --- | --- |
|  | **N** | **%** | **N** | **%** | **N** | **%** | **N** | **%** | **N** | **%** | **N** | | **%** | | | | | | |  | | | | | | |  |
| **SL >21 days** |  |  |  |  |  |  |  |  |  |  |  | |  | | | | | | | 0.025 | | | | | | |  |
| No | 323 | 51.6 | 115 | 57.2 | 84 | 57.9 | 47 | 46.1 | 47 | 43.9 | 30 | | 42.3 | | | | | | |  | | | | | | |  |
| Yes | 303 | 48.4 | 86 | 42.8 | 61 | 42.1 | 55 | 53.9 | 60 | 56.1 | 41 | | 57.7 | | | | | | |  | | | | | | |  |
| **SL duration (months) for women with SL >21 days (n=303)** |  |  |  |  |  |  |  |  |  |  |  | |  | | | | | | |  | | | | | | |  |
| Median, IQR | 9.8 | 4.0-16.7 | 6.1 | 2.2-10.7 | 4.6 | 1.6-9.8 | 12.1 | 8.8-18.2 | 13.2 | 9.7-19.7 | 16.8 | | 10.4-22.6 | | | | | | | <0.001^b^ | | | | | | |  |
| Mean, SD | 11.6 | 9.5 | 7.6 | 8.2 | 7.6 | 8.2 | 14.6 | 9.8 | 15.4 | 8.7 | 16.3 | | 8.8 | | | | | | |  | | | | | | |  |
| **Age** |  |  |  |  |  |  |  |  |  |  |  | |  | | | | | | |  | | | | | | |  |
| Median, IQR | 49.1 | 44.0-54.3 | 49.9 | 45.9-54.8 | 50.6 | 46.8-55.6 | 47.2 | 41.8-52.5 | 47.2 | 42.2-52.0 | 44.4 | | 40.2-50.5 | | | | | | | <0.001^b^ | | | | | | |  |
| Mean, SD | 48.8 | 7.4 | 50.2 | 6.7 | 50.9 | 6.1 | 47.4 | 7.8 | 47.2 | 8.2 | 44.9 | | 7.5 | | | | | | |  | | | | | | |  |
| **Household composition** |  |  |  |  |  |  |  |  |  |  |  | |  | | | | | | | 0.002 | | | | | | |  |
| In couple without <18-year-old children | 241 | 38.5 | 81 | 40.3 | 65 | 44.8 | 39 | 38.2 | 35 | 32.7 | 21 | | | 29.6 | | | | | | |  | | | | | | |
| In couple with <18-year-old children | 170 | 27.2 | 51 | 25.4 | 25 | 17.2 | 29 | 28.4 | 35 | 32.7 | 30 | | | 42.3 | | | | | | |  | | | | | | |
| Single without <18-year-old children | 161 | 25.7 | 56 | 27.9 | 43 | 29.7 | 27 | 26.5 | 27 | 25.2 | 8 | | 11.3 | | | | | | |  | | | | | | |  |
| Single with <18-year-old children | 54 | 8.6 | 13 | 6.5 | 12 | 8.3 | 7 | 6.9 | 10 | 9.3 | 12 | | 16.9 | | | | | | |  | | | | | | |  |
| **Household income^c^** |  |  |  |  |  |  |  |  |  |  |  | |  | | | | | | | 0.369 | | | | | | |  |
| Low income | 133 | 21.2 | 38 | 18.9 | 25 | 17.2 | 25 | 24.5 | 26 | 24.3 | 19 | | 26.8 | | | | | | |  | | | | | | |  |
| Middle income | 253 | 40.4 | 75 | 37.3 | 61 | 42.1 | 40 | 39.2 | 47 | 43.9 | 30 | | 42.3 | | | | | | |  | | | | | | |  |
| High income | 240 | 38.3 | 88 | 43.8 | 59 | 40.7 | 37 | 36.3 | 34 | 31.8 | 22 | | 31.0 | | | | | | |  | | | | | | |  |
| **Education level** |  |  |  |  |  |  |  |  |  |  |  | |  | | | | | | |  | | | | | | |  |
| Primary and secondary education | 248 | 39.6 | 80 | 39.8 | 59 | 40.7 | 49 | 48.0 | 41 | 38.3 | 19 | | 26.8 | | | | | | |  | | | | | | |  |
| Higher education | 378 | 60.4 | 121 | 60.2 | 86 | 59.3 | 53 | 52.0 | 66 | 61.7 | 52 | | 73.2 | | | | | | |  | | | | | | |  |
| **Occupational category** |  |  |  |  |  |  |  |  |  |  |  | |  | | | | | | |  | | | | | | |  |
| Intellectual professionals/managers | 144 | 23.0 | 46 | 22.9 | 34 | 23.5 | 22 | 21.6 | 27 | 25.2 | 14 | | 19.7 | | | | | | |  | | | | | | |  |
| Employees/clerks | 252 | 40.3 | 80 | 39.8 | 56 | 38.6 | 48 | 47.1 | 39 | 36.4 | 29 | | 40.8 | | | | | | |  | | | | | | |  |
| Intermediate profession/technicians | 207 | 33.1 | 69 | 34.3 | 50 | 34.5 | 27 | 26.5 | 37 | 34.6 | 24 | | 33.8 | | | | | | |  | | | | | | |  |
| Skilled/unskilled manual workers | 23 | 3.7 | 6 | 3.0 | 5 | 3.4 | 4 | 3.9 | 4 | 3.7 | 4 | | 5.6 | | | | | | |  | | | | | | |  |
| **Total mastectomy** |  |  |  |  |  |  |  |  |  |  |  | |  | | | | | | | <0.001 | | | | | | |  |
| No | 467 | 74.6 | 145 | 72.1 | 136 | 93.8 | 66 | 64.7 | 76 | 71.0 | 44 | | 62.0 | | | | | | |  | | | | | | |  |
| Yes | 159 | 25.4 | 56 | 27.9 | 9 | 6.2 | 36 | 35.3 | 31 | 29.0 | 27 | | 38.0 | | | | | | |  | | | | | | |  |
| **Breast-conservative surgery** |  |  |  |  |  |  |  |  |  |  |  | |  | | | | | | | <0.001 | | | | | | |  |
| No | 124 | 19.8 | 32 | 15.9 | 15 | 10.3 | 26 | 25.5 | 31 | 29.0 | 20 | | 28.2 | | | | | | |  | | | | | | |  |
| Yes | 502 | 80.2 | 169 | 84.1 | 130 | 89.7 | 76 | 74.5 | 76 | 71.0 | 51 | | 71.8 | | | | | | |  | | | | | | |  |
| **Axillary lymph node dissection** |  |  |  |  |  |  |  |  |  |  |  | |  | | | | | | | <0.001 | | | | | | |  |
| No | 264 | 42.2 | 85 | 42.3 | 105 | 72.4 | 12 | 11.8 | 50 | 46.7 | 12 | | 16.9 | | | | | | |  | | | | | | |  |
| Yes | 362 | 57.8 | 116 | 57.7 | 40 | 27.6 | 90 | 88.2 | 57 | 53.3 | 59 | | 83.1 | | | | | | |  | | | | | | |  |
| **Breast reconstruction** |  |  |  |  |  |  |  |  |  |  |  | |  | | | | | | | <0.001 | | | | | | |  |
| No | 530 | 84.7 | 149 | 74.1 | 141 | 97.2 | 86 | 84.3 | 98 | 91.6 | 56 | | 78.9 | | | | | | |  | | | | | | |  |
| Yes | 96 | 15.3 | 52 | 25.9 | 4 | 2.8 | 16 | 15.7 | 9 | 8.4 | 15 | | 21.1 | | | | | | |  | | | | | | |  |
| **Chemotherapy** |  |  |  |  |  |  |  |  |  |  |  | |  | | | | | | | <0.001 | | | | | | |  |
| No | 342 | 54.6 | 199 | 99.0 | 143 | 98.6 | 0 | 0.0 | 0 | 0.0 | 0 | | 0.0 | | | | | | |  | | | | | | |  |
| Yes | 284 | 45.4 | 2 | 1.0 | 2 | 1.4 | 102 | 100.0 | 107 | 100.0 | 71 | | 100.0 | | | | | | |  | | | | | | |  |
| **Radiotherapy** |  |  |  |  |  |  |  |  |  |  |  | |  | | | | | | | <0.001 | | | | | | |  |
| No | 346 | 55.3 | 201 | 100.0 | 0 | 0.0 | 102 | 100.0 | 2 | 1.9 | 41 | | 57.7 | | | | | | |  | | | | | | |  |
| Yes | 280 | 44.7 | 0 | 0.0 | 145 | 100.0 | 0 | 0.0 | 105 | 98.1 | 30 | | 42.3 | | | | | | |  | | | | | | |  |
| **Hormone therapy** |  |  |  |  |  |  |  |  |  |  |  | |  | | | | | | | <0.001 | | | | | | |  |
| No | 198 | 31.6 | 88 | 43.8 | 38 | 26.2 | 29 | 28.4 | 18 | 16.8 | 25 | | 35.2 | | | | | | |  | | | | | | |  |
| Yes | 428 | 68.4 | 113 | 56.2 | 107 | 73.8 | 73 | 71.6 | 89 | 83.2 | 46 | | 64.8 | | | | | | |  | | | | | | |  |
| **Antidepressant/anxiolytic drug delivery** |  |  |  |  |  |  |  |  |  |  |  | |  | | | | | | | <0.001 | | | | | | |  |
| No | 243 | 38.8 | 98 | 48.8 | 63 | 43.4 | 31 | 30.4 | 30 | 28.0 | 21 | | 29.6 | | | | | | |  | | | | | | |  |
| Yes | 383 | 61.2 | 103 | 51.2 | 82 | 56.6 | 71 | 69.6 | 77 | 72.0 | 50 | | 70.4 | | | | | | |  | | | | | | |  |
| **Antalgic drug delivery** |  |  |  |  |  |  |  |  |  |  |  | |  | | | | | | | <0.001 | | | | | | |  |
| No | 229 | 36.6 | 103 | 51.2 | 52 | 35.9 | 41 | 40.2 | 15 | 14.0 | 18 | | 25.4 | | | | | | |  | | | | | | |  |
| Yes | 397 | 63.4 | 98 | 48.8 | 93 | 64.1 | 61 | 59.8 | 92 | 86.0 | 53 | | 74.6 | | | | | | |  | | | | | | |  |
|  | | | | | | | | | | | |  | |  |  |  |  |  |  |  | |  |  |  |  |  | |

^a^Chi-square and Fisher’s exact tests. ^b^Wilcoxon rank test. ^c^Low income: Income <1,500€ for one or <2,800€ for two or more income contributors; Middle income: Income between 1,500€ and 2,000€ for one or between 2,800€ and 4,100€ for two or more income contributors; High income: Income >2,000€ for one or >4,100€ for two or more income contributors

S: “Surgery”; SR: ”Surgery and radiotherapy”; SC: “Surgery and chemotherapy”; SCR: ”Surgery, chemotherapy and radiotherapy”; SLC: “Surgery and long chemotherapy”.

SL: Sick leave identified in the SNDS

**Table S2. Characteristics and treatments of women with a sick leave identified in the SNDS**

|  | |  | |  | |  | |  | |  |  | |  |  |  |
| --- | --- | --- | --- | --- | --- | --- | --- | --- | --- | --- | --- | --- | --- | --- | --- |
|  | **Women with SL**  **N = 349** | | | | **SL duration ≤21 days**  **N=46** | | | | **SL duration >21 days**  **N=303** | | | | | |  |
|  | N | | % | | N | | % | | N | | | % | | | p^a^ |
| **Age (years)** |  | |  | |  | |  | |  | | |  | | |  |
| Median, IQR | 49.1 | | 44.4-53.5 | | 50.6 | | 44.8-54.4 | | 48.7 | | | 44.4-53.4 | | | 0.190^b^ |
| Mean, SD | 48.5 | | 7.2 | | 49.6 | | 8,4 | | 48.4 | | | 7,0 | | |  |
| **Household composition** |  | |  | |  | |  | |  | | |  | | | 0.981 |
| Single without <18-year-old children | 88 | | 25.2 | | 11 | | 23.9 | | 77 | | | 25.4 | | |  |
| Single with <18-year-old children | 33 | | 9.5 | | 4 | | 8.7 | | 29 | | | 6.6 | | |  |
| In couple without <18-year-old children | 140 | | 40.1 | | 18 | | 39.1 | | 122 | | | 40.3 | | |  |
| In couple with <18-year-old children | 88 | | 25.2 | | 13 | | 28.3 | | 75 | | | 24.8 | | |  |
| **Household income^c^** |  | |  | |  | |  | |  | | |  | | | 0.055 |
| Low income | 98 | | 28.1 | | 7 | | 15.2 | | 91 | | | 30.0 | | |  |
| Middle income | 143 | | 41.0 | | 19 | | 41.3 | | 124 | | | 40.9 | | |  |
| High income | 108 | | 30.9 | | 20 | | 43.5 | | 88 | | | 29.0 | | |  |
| **Education level** |  | |  | |  | |  | |  | | |  | | | 0.103 |
| Primary and secondary education | 168 | | 48.1 | | 17 | | 37.0 | | 151 | | | 49.8 | | |  |
| Highr education | 181 | | 51.9 | | 29 | | 63.0 | | 152 | | | 50.2 | | |  |
| **Occupational category at diagnosis** |  | |  | |  | |  | |  | | |  | | | 0.534 |
| Intellectual professionals/managers | 70 | | 20.1 | | 13 | | 28.3 | | 57 | | | 18.8 | | |  |
| Employees/clerks | 183 | | 52.4 | | 22 | | 47.8 | | 161 | | | 53.1 | | |  |
| Intermediate profession/technicians | 77 | | 22.1 | | 9 | | 19.6 | | 68 | | | 22.4 | | |  |
| Skilled/unskilled manual workers | 19 | | 5.4 | | 2 | | 4.3 | | 17 | | | 5.6 | | |  |
| **Total mastectomy** |  | |  | |  | |  | |  | | |  | | | 0.221 |
| No | 263 | | 75.4 | | 38 | | 82.6 | | 225 | | | 74.3 | | |  |
| Yes | 86 | | 24.6 | | 8 | | 17.4 | | 78 | | | 25.7 | | |  |
| **Breast-conservative surgery** |  | |  | |  | |  | |  | | |  | | | 0.160 |
| No | 64 | | 18.3 | | 5 | | 10.9 | | 59 | | | 19.5 | | |  |
| Yes | 285 | | 18.7 | | 41 | | 89.1 | | 244 | | | 80.5 | | |  |
| **Axillary lymph node dissection** |  | |  | |  | |  | |  | | |  | | | 0.025 |
| No | 130 | | 37.2 | | 24 | | 52.2 | | 106 | | | 35.0 | | |  |
| Yes | 219 | | 62.8 | | 22 | | 47.8 | | 197 | | | 65.0 | | |  |
| **Breast reconstruction** |  | |  | |  | |  | |  | | |  | | | 0.826 |
| No | 292 | | 83.7 | | 39 | | 84.8 | | 253 | | | 83.5 | | |  |
| Yes | 57 | | 16.3 | | 7 | | 15.2 | | 50 | | | 16.5 | | |  |
| **Chemotherapy** |  | |  | |  | |  | |  | | |  | | | 0.003 |
| No | 180 | | 51.6 | | 33 | | 71.7 | | 147 | | | 48.5 | | |  |
| Yes | 169 | | 48.4 | | 13 | | 28.3 | | 156 | | | 51.5 | | |  |
| **Radiotherapy** |  | |  | |  | |  | |  | | |  | | | 0.619 |
| No | 193 | | 55.3 | | 27 | | 58.7 | | 166 | | | 54.8 | | |  |
| Yes | 156 | | 44.7 | | 19 | | 41.3 | | 137 | | | 45.2 | | |  |
| **Hormone therapy** |  | |  | |  | |  | |  | | |  | | | 0.024 |
| No | 109 | | 31.2 | | 21 | | 45.7 | | 88 | | | 29.0 | | |  |
| Yes | 240 | | 68.8 | | 25 | | 54.3 | | 215 | | | 71.0 | | |  |
| **Antidepressant/anxiolytic drug delivery** |  | |  | |  | |  | |  | | |  | | | 0.001 |
| No | 131 | | 37.5 | | 27 | | 58.7 | | 104 | | | 34.3 | | |  |
| Yes | 218 | | 62.5 | | 19 | | 41.3 | | 199 | | | 65.7 | | |  |
| **Antalgic drug delivery** |  | |  | |  | |  | |  | | |  | | | 0.440 |
| No | 119 | | 34.1 | | 18 | | 39.1 | | 101 | | | 33.3 | | |  |
| Yes | 230 | | 65.9 | | 28 | | 60.9 | | 202 | | | 66.7 | | |  |
| **Care pathway^d^** |  | |  | |  | |  | |  | | |  | | | 0.052 |
| Pattern S | 105 | | 30.1 | | 19 | | 41.3 | | 86 | | | 28.4 | | |  |
| Pattern SR | 75 | | 21.5 | | 14 | | 30.4 | | 61 | | | 20.1 | | |  |
| Pattern SC | 61 | | 17.5 | | 6 | | 13.0 | | 55 | | | 18.2 | | |  |
| Pattern SCR | 63 | | 18.1 | | 3 | | 6.5 | | 60 | | | 19.8 | | |  |
| Pattern SLC | 45 | | 12.9 | | 4 | | 8.7 | | 41 | | | 13.5 | | |  |

^a^Chi-square and Fisher’s exact tests; ^b^Wilcoxon rank test.

^c^Low income: Income <1,500€ for one or <2,800€ for two or more income contributors; Middle income: Income between 1,500€ and 2,000€ for one or between 2,800€ and 4,100€ for two or more income contributors; High income: Income >2,000€ for one or >4,100€ for two or more income contributors

^d^S: “Surgery”; SR: ”Surgery and radiotherapy”; SC: “Surgery and chemotherapy”; SCR: ”Surgery, chemotherapy and radiotherapy”; SLC: “Surgery and long chemotherapy”.

SL: Sick leave


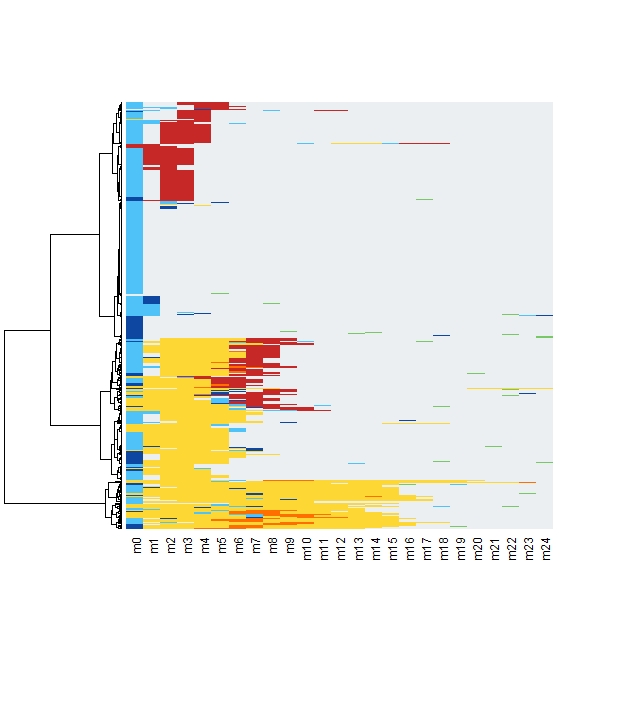


**
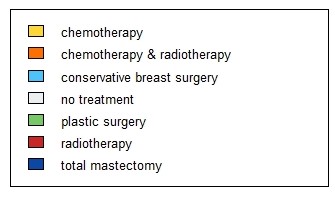
**

**Fig S1: Dendrogram showing the hierarchical clustering of different BC treatment sequences using the optimal matching distances, Ward method.**


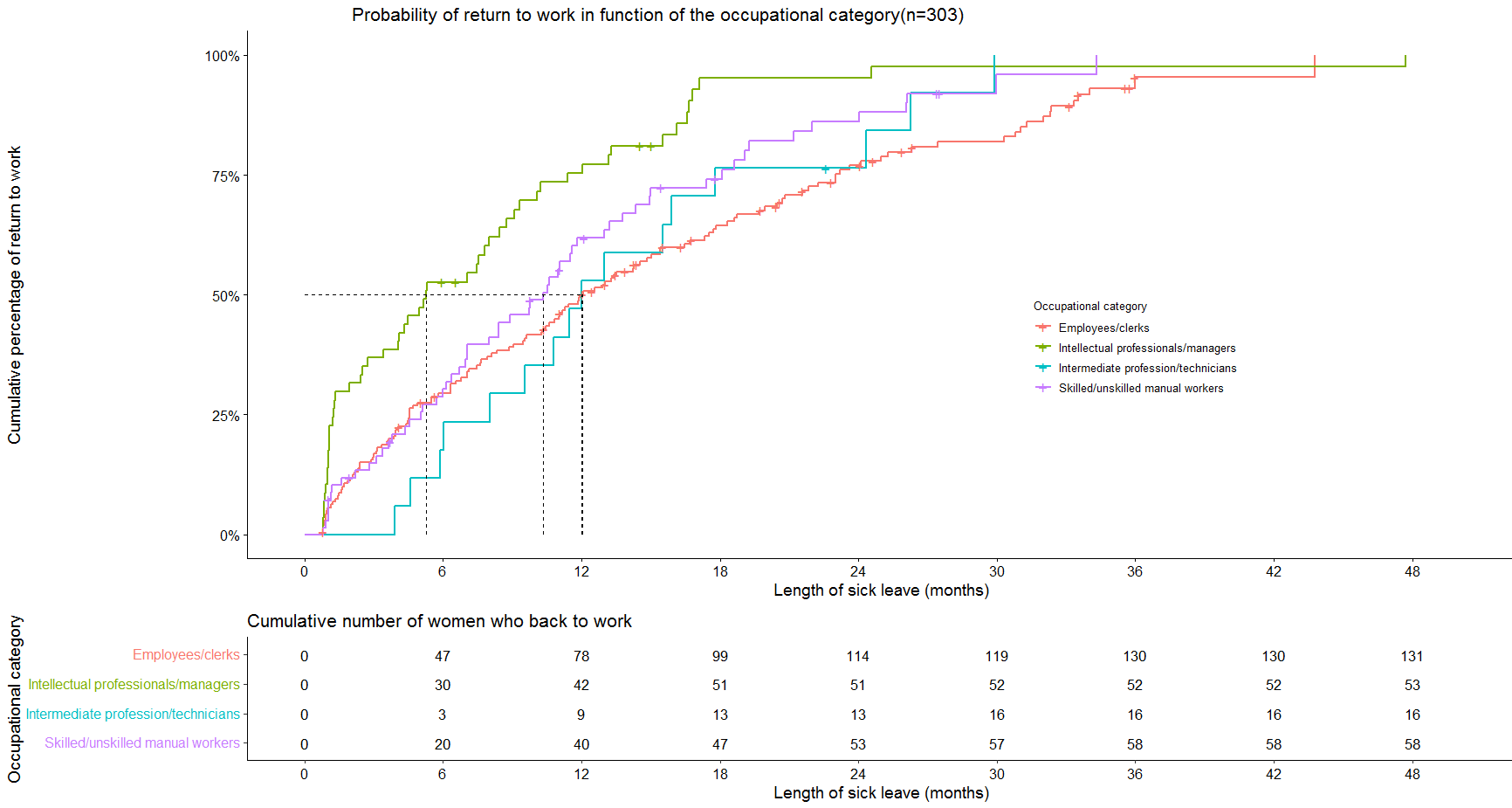


Median follow-up time in months (IQR):33.5 (21.6;36.0) ; Median survival time in months (IQR):9.8 (4.0;16.73)

**Fig S2. Cumulative incidence of return to work according to the occupational category.**

**Table S3: BC treatments associated with the likelihood of return to work, univariate analyses and after adjustment for other BC treatments only (model 1), for BC treatments and drug deliveries (model 2), for BC treatments, socioeconomic characteristics, and age (model 3) (n=303)**

|  | **Univariate analysis** |  | **Model 1** |  | **Model 2** |  | **Model 3** |  |
| --- | --- | --- | --- | --- | --- | --- | --- | --- |
|  |  | **p^a^** | **HR (95% CI)** | **p^a^** | **HR (95% CI)** | **p^a^** | **HR (95% CI)** | **p^a^** |
| **Breast surgery^b^** |  |  |  |  |  |  |  |  |
| **No** | Reference | - | Reference | - | Reference | - | Reference | - |
| **Yes** | 0.09 (0.02-0.38) | 0.001 | 0.09 (0.02-0.44) | 0.003 | 0.10 (0.02-0.44) | 0.003 | 0.08 (0.02-0.38) | 0.002 |
| **Chemotherapy** |  |  |  |  |  |  |  |  |
| **No** | Reference | - | Reference | - | Reference | - | Reference | - |
| **Yes** | 0.16 (0.07-0.36) | <0.001 | 0.13 (0.05-0.33) | <0.001 | 0.13 (0.05-0.32) | <0.001 | 0.08 (0.03-0.21) | <0.001 |
| **Radiotherapy** |  |  |  |  |  |  |  |  |
| **No** | Reference | - | Reference | - | Reference | - | Reference | - |
| **Yes** | 0.52 (0.28-0.99) | 0.045 | 0.41 (0.22-0.76) | 0.005 | 0.41 (0.22-0.76) | 0.005 | 0.36 (0.19-0.67) | 0.001 |
| **Axillary lymph node dissection** |  |  |  |  |  |  |  |  |
| **No** | Reference | - | Reference | - | Reference | - | Reference | - |
| **Yes** | 0.06 (0.01-0.48) | 0.009 | 0,09 (0.01-1,21) | 0.069 | 0.09 (0.01-1.16) | 0.065 | 0.17 (0.01-2.14) | 0.171 |
| **Hormone therapy** |  |  |  |  |  |  |  |  |
| **No** | Reference | - | Reference | - | Reference | - | Reference | - |
| **Yes** | 1.12 (0.75-1.66) | 0.584 | 0.83 (0.56-1.24) | 0.357 | 0.82 (0.56-1.19) | 0.300 | 0.88 (0.62-1.27) | 0.505 |
| **Antidepressant/anxiolytic drugs** |  |  |  |  |  |  |  |  |
| **No** | Reference | - | - | - | Reference | - | - | - |
| **Yes** | 0.80 (0.58-1.13) | 0.205 | - | - | 0.79 (0.56-1.13) | 0.199 | - | - |
| **Antalgic drugs** |  |  |  |  |  |  |  |  |
| **No** | Reference | - | - | - | Reference | - | - | - |
| **Yes** | 0.74 (0.56-0.97) | 0.028 | - | - | 0.80 (0.63-1.03) | 0.085 | - | - |
| **Age (years)** |  |  |  |  |  |  |  |  |
| 20-44 | Reference | - | - | - | - | - | Reference | - |
| 45-49 | 0.89 (0.56-1.42) | 0.626 | - | - | - | - | 0.82 (0.50-1.37) | 0.455 |
| 50-55 | 0.92 (0.54-1.56) | 0.752 | - | - | - | - | 1.12 (0.66-1.92) | 0.673 |
| >55 | 0.65 (0.42-1.01) | 0.053 | - | - | - | - | 0.49 (0.26-0.93) | 0.030 |
| **Household income**^c^ |  |  |  |  |  |  |  |  |
| Low income | Reference | - | - | - | - | - | Reference | - |
| Middle income | 1.34 (0.89-2.01) | 0.157 | - | - | - | - | 1.14 (0.74-1.75) | 0.546 |
| High income | 3.04 (1.71-5.41) | <0.001 | - | - | - | - | 2.86 (1.35-6.07) | 0.006 |
| **Household composition** |  |  |  |  |  |  |  |  |
| In couple without <18-year-old children | Reference | - | - | - | - | - | Reference | - |
| In couple with <18-year-old children | 1.48 (0.97-2.26) | 0.067 | - | - | - | - | 0.94 (0.53-1.65) | 0.821 |
| Single without <18-year-old children | 0.63 (0.28-1.45) | 0,281 | - | - | - | - | 0.66 (0.31-1.42) | 0.284 |
| Single with <18-year-old children | 0.98 (0.61-1.58) | 0,938 | - | - | - | - | 0.64 (0.37-1.12) | 0.121 |
| **Education level** |  |  |  |  |  |  |  |  |
| Primary and secondary education | Reference | - | - | - | - | - | Reference | - |
| Higher education | 1.21 (0.82-1.80) | 0,331 | - | - | - | - | 0.98 (0.59-1.64) | 0.945 |

^a^Log-rank test.

^b^The two breast surgery types (total mastectomy and breast-conserving surgery) were combined into a single “breast surgery” variable due to the limited number of women in the two initial categories.

^c^Low income: <1,500€ if one contributor or <2,800€ if two or more contributors in the household; Middle income: between 1,500€ and 2,000€ if one contributor or between 2,800€ and 4,100€ if two or more contributors in the household; High income: ≥2,000€ if one or ≥ 4,100€ if two or more contributors in the household.
